# Author Correction: Sequence terminus dependent PCR for site-specific mutation and modification detection

**DOI:** 10.1038/s41467-023-37301-6

**Published:** 2023-03-20

**Authors:** Gaolian Xu, Hao Yang, Jiani Qiu, Julien Reboud, Linqing Zhen, Wei Ren, Hong Xu, Jonathan M. Cooper, Hongchen Gu

**Affiliations:** 1 School of Biomedical Engineering/Med-X Research Institute, Shanghai Jiao Tong University, Shanghai, 200030 China; 2 Division of Biomedical Engineering, University of Glasgow, G12 8LT Glasgow, United Kingdom

**Keywords:** Cancer epigenetics, PCR-based techniques, Genetic testing, DNA methylation, Mutation

Correction to: *Nature Communications* 10.1038/s41467-023-36884-4, published online 01 March 2023

The original version of this Article contained an error in Fig. 1, in which panel b was missing, and panels c–e were incorrectly labelled as b–d respectively. The correct version of Fig. 1 is:


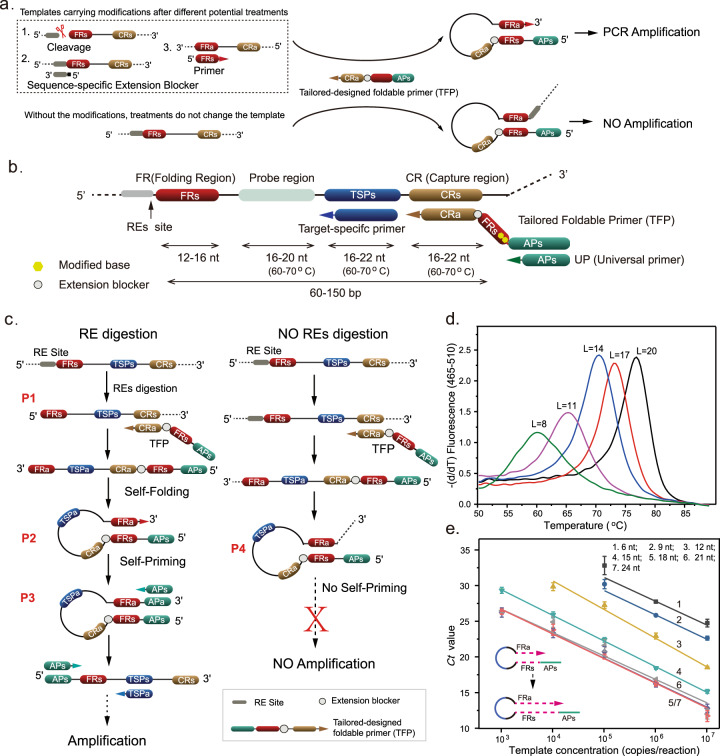


which replaces the previous incorrect version:


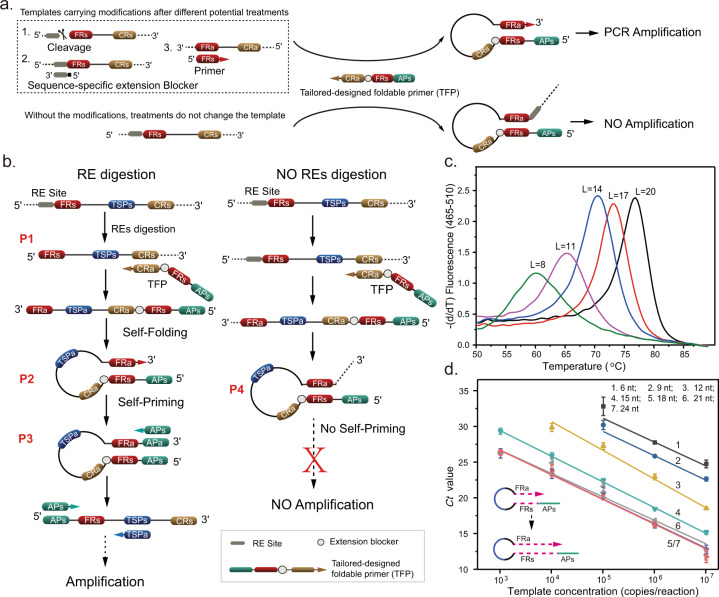


This has been corrected in both the PDF and HTML versions of the Article.

