# Supplementary material for: Sequence terminus dependent PCR for site-specific mutation and modification detection
Source: Nat Commun. 2023 Mar 1;14:1169. doi: 10.1038/s41467-023-36884-4 (PMC9978023; doi:10.1038/s41467-023-36884-4)
Supplement: Supplementary file 1 — Supplementary Information [file 41467_2023_36884_MOESM1_ESM.pdf]

Sequence terminus dependent PCR for site-specific mutation and modification detection

Gaolian Xu,[1†] Hao Yang,[1†] Jiani Qiu, [1†] Julien Reboud[2], Linqing Zhen[1], Wei Ren[1], Hong Xu,[1*] Jonathan M. Cooper,[2*] Hongchen Gu[1*]

1, School of Biomedical Engineering/Med-X Research Institute, Shanghai Jiao Tong University, Shanghai 200030, China.
2. Division of Biomedical Engineering, University of Glasgow, G12 8LT Glasgow, United Kingdom;
These authors (†) contributed equally to this work. Correspondence should be addressed to Hong Xu (xuhong@sjtu.edu.cn), Jonathan M. Cooper (Jon.Cooper@glasgow.ac.uk) or Hongchen Gu (hcgu@sjtu.edu.cn).

Supplementary Information

Supplementary Note 1. Glossary of Terms

Specific Terminal Mediated Polymerase Chain Reaction	STEM-PCR
Allele-specific (AS) quantitative (q)PCR	AS-qPCR
Restriction enzyme	RE
PCR-restriction fragment length polymorphism	PCR-RFLP
Methylation sensitive restriction enzyme	MSRE
Helper-dependent chain reaction	HDCR
Methylation-dependent restriction endonuclease	MDRE
Peptide nucleic acid	PNA
Tailored-designed foldable primer	TFP
Capture region	CR
Artificial primer	AP
Folding region	FR
Colorectal cancer	CRC
Bisulfite-PCR	BS-PCR
Methylation-specific PCR	MSP
Bridge primer	BP
Secreted Frizzled Related Protein 2	SFRP2

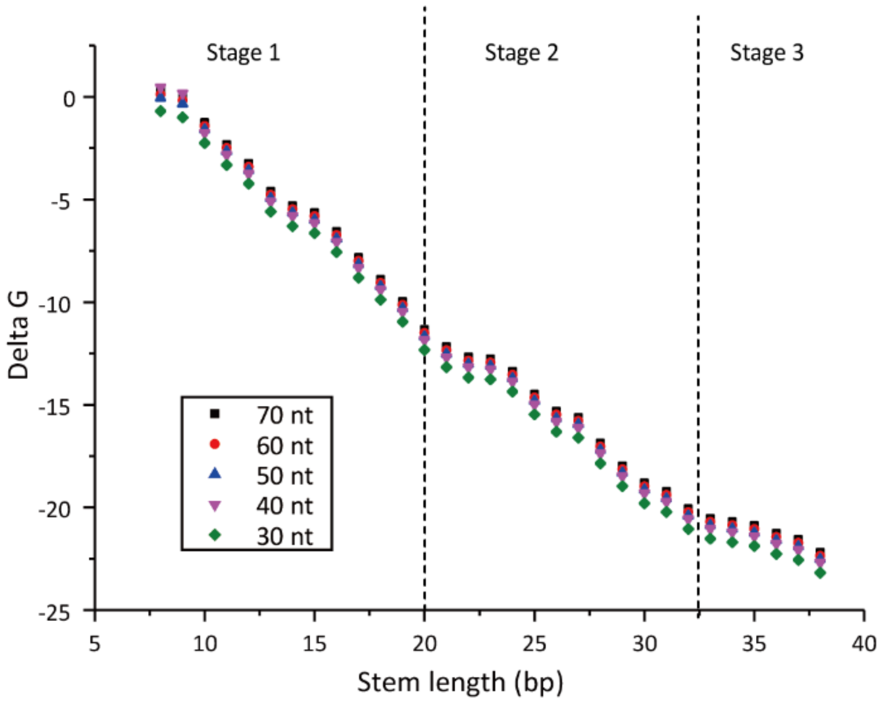

Supplementary Figure 1. Comparison of Tm with hairpin structure with different loop sizes from 70-30 nt. (1). 70 bp (black square); 2. 60 bp (red circle); 3. 50 bp (blue up triangle); 4. 40 bp (magenta down triangle); 5. 30 bp (olive diamond). All the data were calculated at a given reaction environment: 65°C with 1.5 mM Mg^{2+}. We used IDT software to analyse the relationship between the length of FR and self-folding stability of P2 using sequences with different loop size. Given the reaction environment at 65°C with 1.5 mM Mg^{2+}, the difference in Gibbs free energy as the loop size changed from 30-70 nt was not significant. ΔG decreases as the stem length increases, with three main stages. The slopes of linear fits within each stage decreased from 0.998, 0.758 and 0.283 (in absolute value), indicating a weaker dependence between stem length and self-folding of hairpin structure as stem length increasing. This may be due to the average effect of stacking energies of each base pair, which would gradually decrease with the increasing in dsDNA length. Source data are provided as a Source Data file.

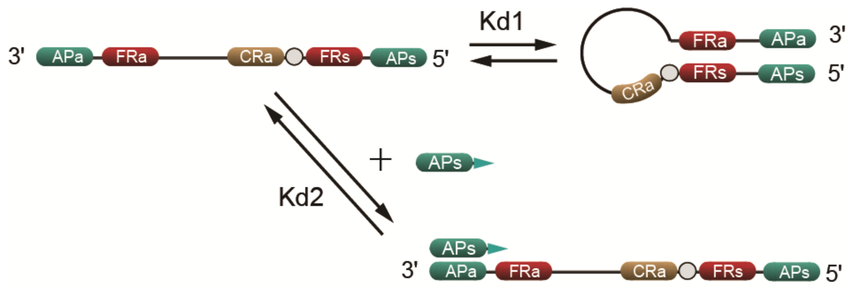

Supplementary Figure 2. Two-state approach for analyses the reaction kinetics of P2 with different states.

Supplementary Note 2 - Reaction kinetics analysis of P3 state

In the initial step of amplification, APs hybridizes to the stem region of P3 to generate a linear product. This state depends on the competition between self-folding state and hetero-hybridization with APs, in turn influenced by the sequence and size of the P3 loop, the sequence and size of the P3 stem, the salt concentration and reaction temperature. Here, we used a two-state approach to analyse the reaction kinetics of this competition process. The reaction constant (K_{d1}) for the formation of hairpin structure (Supplementary Figure 3) is defined as:

$$K_{d1} = \frac{[C_{Hairpin}]}{[C_{Single\ strand}]} \qquad \text{Eq. 1}$$

While K_{d2} for hybridization between the single strand P2 and APs (Supplementary Figure 3) is defined as:

$$K_{d2} = \frac{[C_{Hybridized}]}{[C_{Single\ strand}][Primer]} \qquad \text{Eq. 2}$$

Given a total concentration of primer, K_{d2} can be converted into:

$$K_{d2} = \frac{[C_{Hybridized}]}{[C_{Single\ strand}]([Primer_{total}]-[C_{Hybridized}])} \qquad \text{Eq. 3}$$

At the linear amplification step,

$$[Primer_{total}] >> [C_{Hybridized}]$$

leading to

$$K_{d2} = \frac{[C_{Hybridized}]}{[C_{Single\ strand}][Primer_{total}]} \qquad \text{Eq. 4}$$

At any given temperature, under an equilibrium state,

$$[C_{Single\ strnad}] = \frac{[C_{Hairpin}]}{K_{d1}} = \frac{[C_{Hybridized}]}{K_{d2}[Primer_{total}]} \qquad \text{Eq. 5}$$

$$\frac{[C_{Hybridized}]}{[C_{Hairpin}]} = \frac{K_{d2}[Primer_{total}]}{K_{d1}} \qquad \text{Eq. 6}$$

K_{d1} and K_{d2} in turn directly depend on the Gibbs free energy change:

$$\Delta G = -RTln(K_d) \qquad \text{Eq. 7}$$

Equation 6 can be converted into:

$$\frac{[C_{Hybridized}]}{[C_{Hairpin}]} = \frac{e^{-\frac{\Delta G2}{RT}}}{e^{-\frac{\Delta G1}{RT}}}[Primer_{total}] \qquad \text{Eq. 8}$$

$$\frac{[C_{Hybridized}]}{[C_{Hairpin}]} = e^{\frac{\Delta G1-\Delta G2}{RT}}[Primer_{total}] \qquad \text{Eq. 9}$$

Given the primer sequence and concentration, the ΔG2 value of the hetero-hybridization state between APs and the stem region of P3 stays constant. Based on equation 9, the ratio of hybridization state and hairpin self-folding state at equilibrium state only depends on the ΔG1 value of complete hairpin structure with self-folding state. Theoretically, the ΔG1 value decreases as the stem length of P3 increased (Supplementary Figure 2), leading to a smaller value of ΔΔG (ΔG1-ΔG2) and low percentage of the molecules in hybridization state. However, as the dependence between the stem length and ΔG1 becomes weaker (Supplementary Figure 2), for example more than 33 bp, leading to the values of C [Hybridized]/ C [Hairpin] fluctuating with a small range once the stem length reaches to a specific plateau size. However, APs hybridizes to the 3' end of P3 and so will have more opportunities to generate a linear product in these cases, due to the high concentration, resulting in hairpins unwinding and generating a linear template.

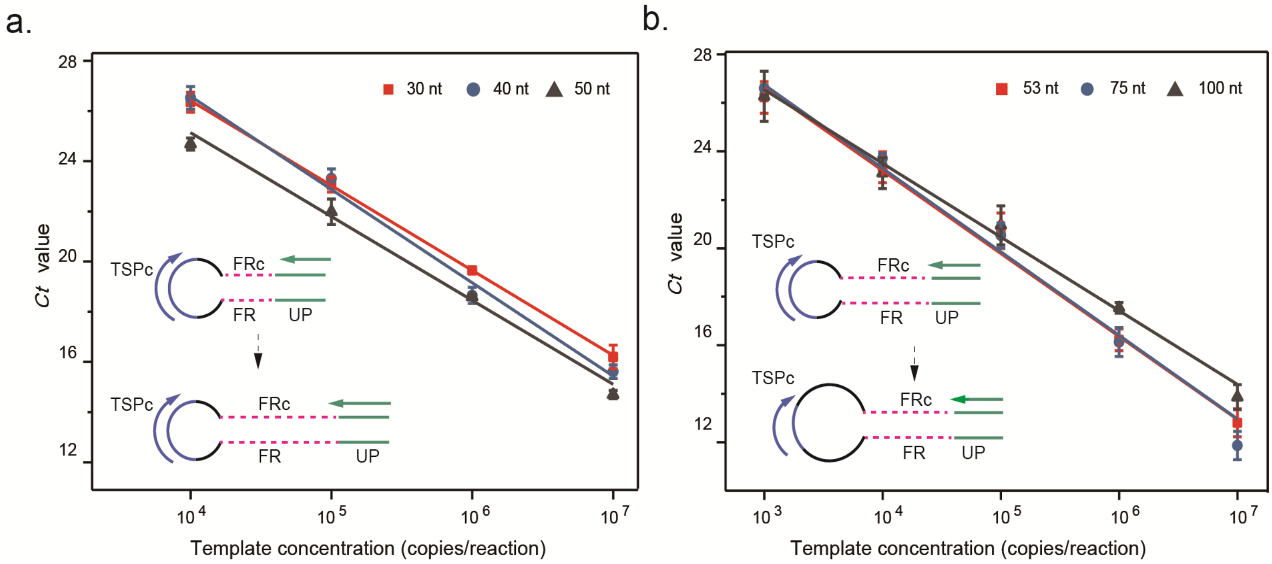

Supplementary Figure 3 (a) Illustration of the effect of different stem lengths, with the loop size at 53 nt. (1). 30 nt (red square); (2). 40 nt (orange triangle); (3). 50 nt (blue circle). *Ct* value as a function of 10x serial diluted templates concentration, from 10^7 to 10^4 copies/reaction. All linear regressions have $R^2>0.99$. (30 nt -0.99, 40nt-0.99, 50nt-0.99), n=3 independent experiments; (b) Illustration of the effect of P3 with different loop lengths, for a 40 nt stem. (1) 53 nt (red square); (2). 75 nt (blue circle); (3). 100 nt (orange triangle). *Ct* value as a function of 10x serial diluted templates concentration, from 10^7 copies/reaction to 10^3 copies/reaction. Data is the average of at least 3 independent experiments and error bars represent the standard deviation. All linear regressions have $R^2>0.99$. (53 nt -0.99, 75nt-0.99, 100nt-0.99). As the stem length of P3 increased from 30 to 50 bp, its structure is a double strand, effectively improving the ability of APs to compete with the self-folding state. Fixing the stem length at 50 nt and changing the loop lengths of P3 from 53-100 nt did not influence the amplification efficiency of STEM-PCR significantly. Hence, the optimized P3 was designed with the loop and stem size about 40-60 nt and FR at 13-16 nt to obtain high efficiency. Source data are provided as a Source Data file.

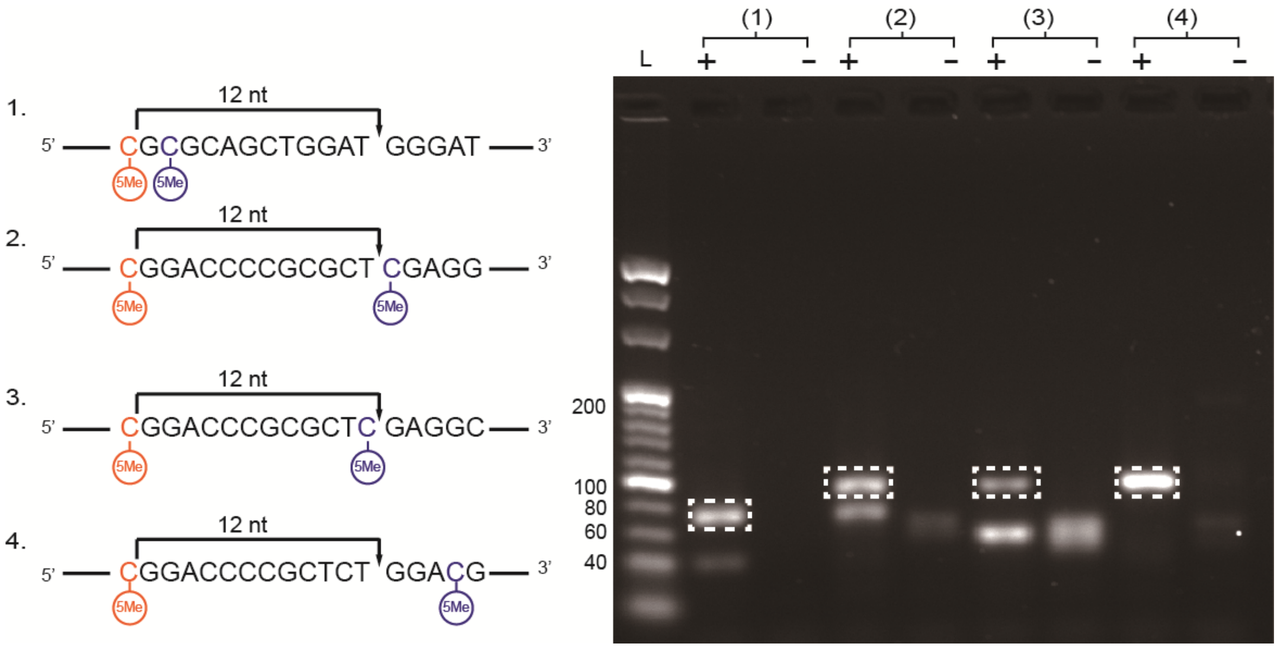

Supplementary Figure 4: (a) Cleavage scenarios with two ^{m}CG at different distances; (1) two ^{m}CpG site at the recognition sequence, with the distance at zero; (2) 12 nucleotides; (3) 11 nucleotides, with the second ^{m}C at cleavage position; (4) Larger than 12 nucleotides; (b) Agarose gel results of STEM-PCR for the different cleavage scenarios. Amplicons of STEM-PCR are marked with white dashed box. (+) denotes a reaction containing the template (100 copies/reaction), and (-) denotes a reaction without template. L: 20 bp ladder. STEM-PCR is specific to distinguish the neighboring modified CpG site even inserted within zero base. It should be noted that the difference in sizes between lanes 1 and (2-4) arises from the fact that the TFP sequence for each scenario was different, leading to different size fragments (i.e. 2-4 also are different). Each experiment was repeated independently at least 3 times with similar results.

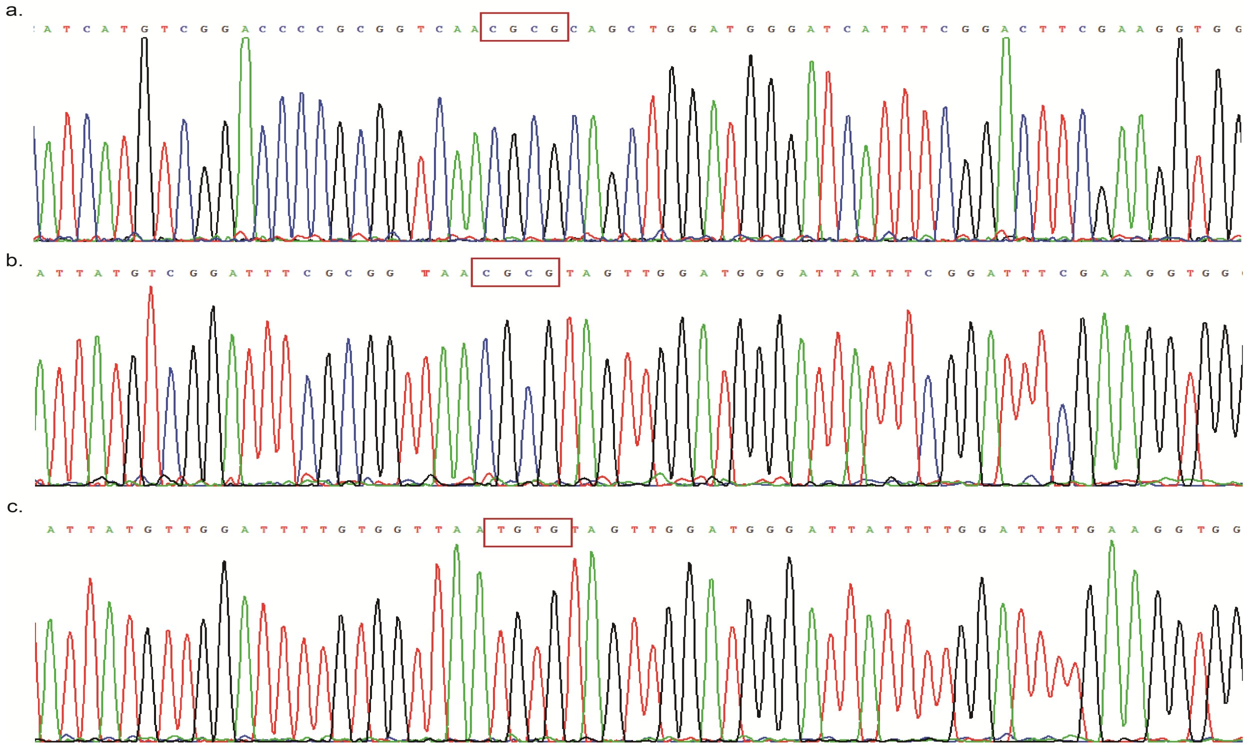

Supplementary Figure 5. DNA sequencing results of Septin9 gene for different treatments: (a) Untreated template; (b) BS-treated methylated template; (c) BS-treated unmethylated template. The red box indicates the location used for STEM-PCR. The same amplification fragments were selected for analyze. Source data are provided as a Source Data file.

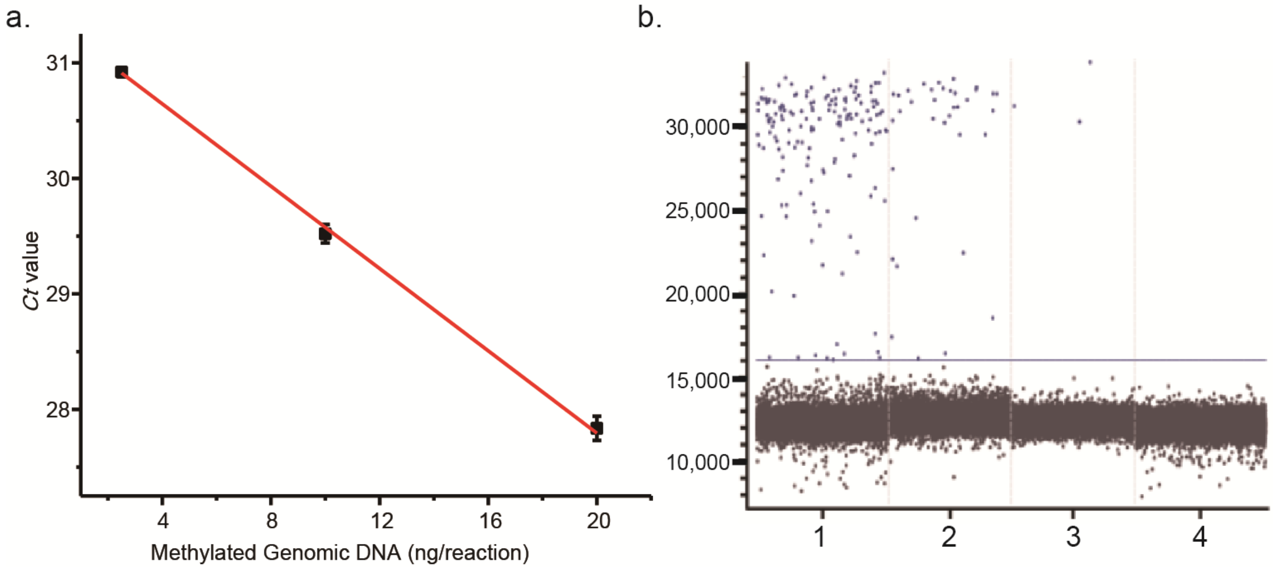

Supplementary Figure 6 (a) Detection of full methylated *SEPTIN9* DNA using commercial HeavyMethyl (red line is linear regression, R^2=0.99). Samples were serially diluted genomic DNA, from 20 ng/reaction to 1.25 ng/reaction, graph showing average of n=3 independent experiments, error bars are standard deviation; (b) ddPCR of serially diluted fully methylated Septin9 DNA treated with GlaI, 1. 240 copies/reaction, 2. 48 copies/reaction, 3. 10 copies/reaction, 4. NC. The copy number in each sample was calculated by fitting a Poisson's distribution: 1. 225 copies/reaction; 2. 56 copies/reaction; 3. 6 copies/reaction and 4. 0 copies/reaction. The sensitivity of HeavyMethyl was about 2.5 ng/reaction (600 copies/reaction) which is ca. 20 times less sensitive than STEM-PCR. Source data are provided as a Source Data file.

a.

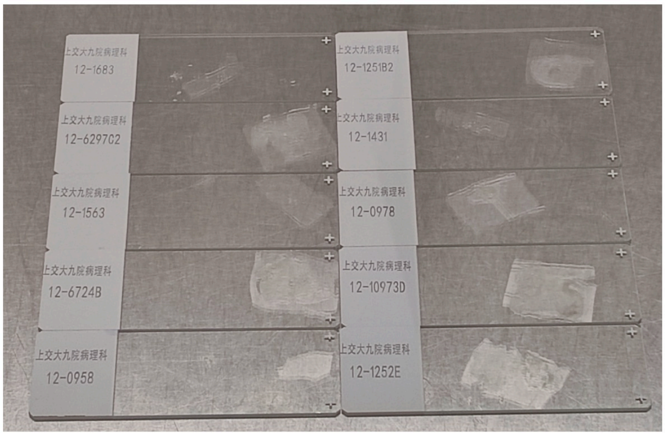

b.

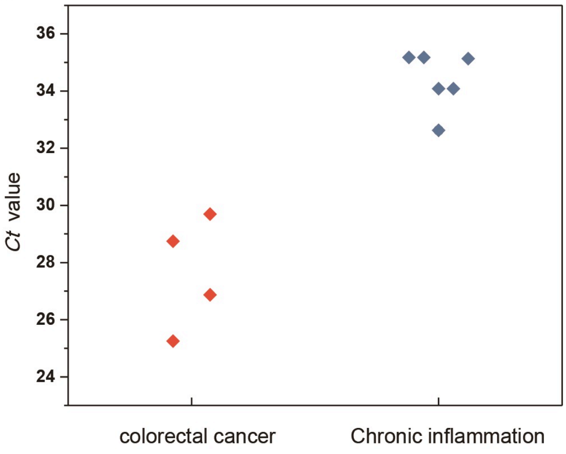

c.

	Pathology	STEM-PCR Ct (average)	Standard deviation
1	Chronic inflammation of the sigmoid mucosa, polypoid growth	34.08	4.19
2	Chronic inflammation of the sigmoid mucosa, polypoid growth	35.18	2.04
3	Rectal adenocarcinoma, size 4*3.5*3 CM	26.87	0.30
4	Rectal adenocarcinoma,size 2*1.5*1.2 CM	29.70	0.64
5	Chronic inflammation of the sigmoid mucosa	34.96	0.41
6	Focal squamous hyperplasia	33.61	3.10
7	Rectal adenocarcinoma,CK+, CK7-, CK19+,EMA+	28.74	0.43
8	Rectal adenocarcinoma, 7.5*5.5*1.5 CM	25.25	0.23
9	Chronic inflammation of the rectal mucosa	35.14	1.72
10	Chronic appendicitis	32.63	2.10

Supplementary Figure 7. (a). Ten years old FFPE samples; (b). Ct value of STEM-PCR using the mechanism described in Figure 1 targeting SEPTIN9, provided with pathology description in (c). All points are shown (4 colorectal cancer samples in red and 8 chronic inflammations in grey). The age and sex of patients was not recorded.

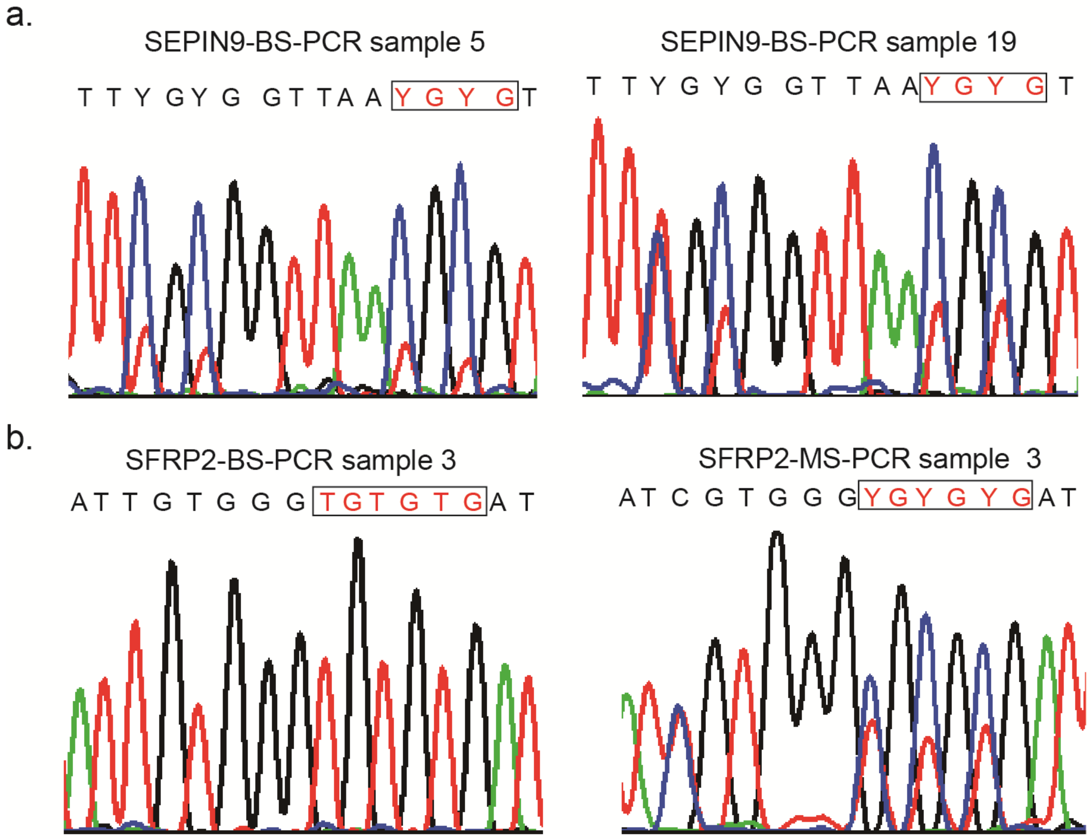

Supplementary Figure 8. Typical BS-PCR and MSP-PCR sequencing results. (a). BSP sequencing results of sample 5 and 19. (b). The BSP and MSP result of sample 3. Source data are provided as a Source Data file.

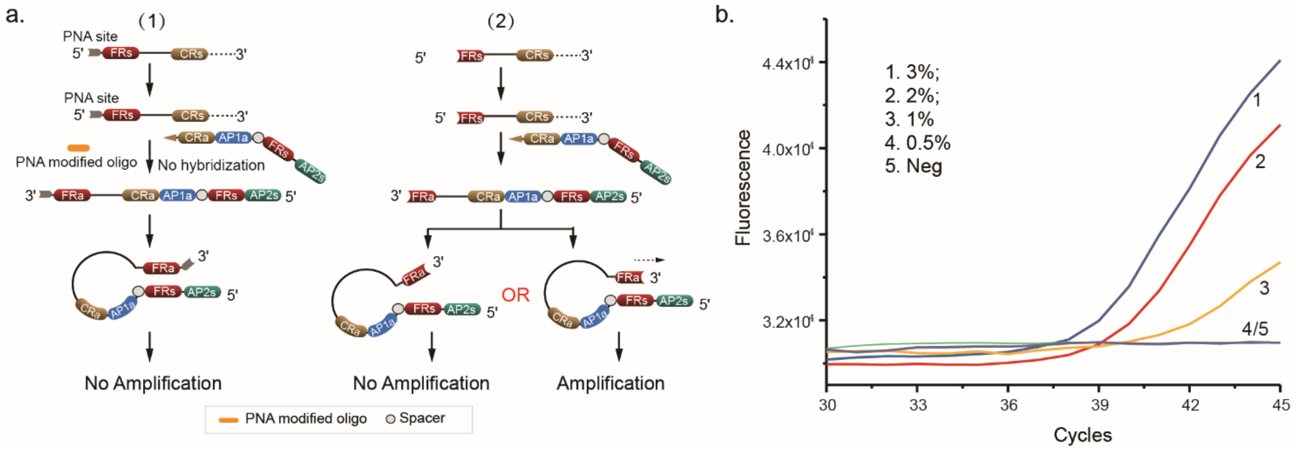

Supplementary Figure 9. Impact of the level of fragmentation of template on STEM-PCR. (a). Different scenarios of fragmentation that affected the outcome of STEM-PCR. 1. Truncated sites of fragmented DNA were distant from the 5' end of FRs; 2. The truncated sites of fragmented DNA lay within FRs region. (b). The sensitivity of PNA mediated STEM-PCR for L858R mutation detection with 3000 copies as background (3% blue, 2% red, 1% yellow, 0.5% green, negative – Neg grey). The limit of detection was below 1%. Source data are provided as a Source Data file.

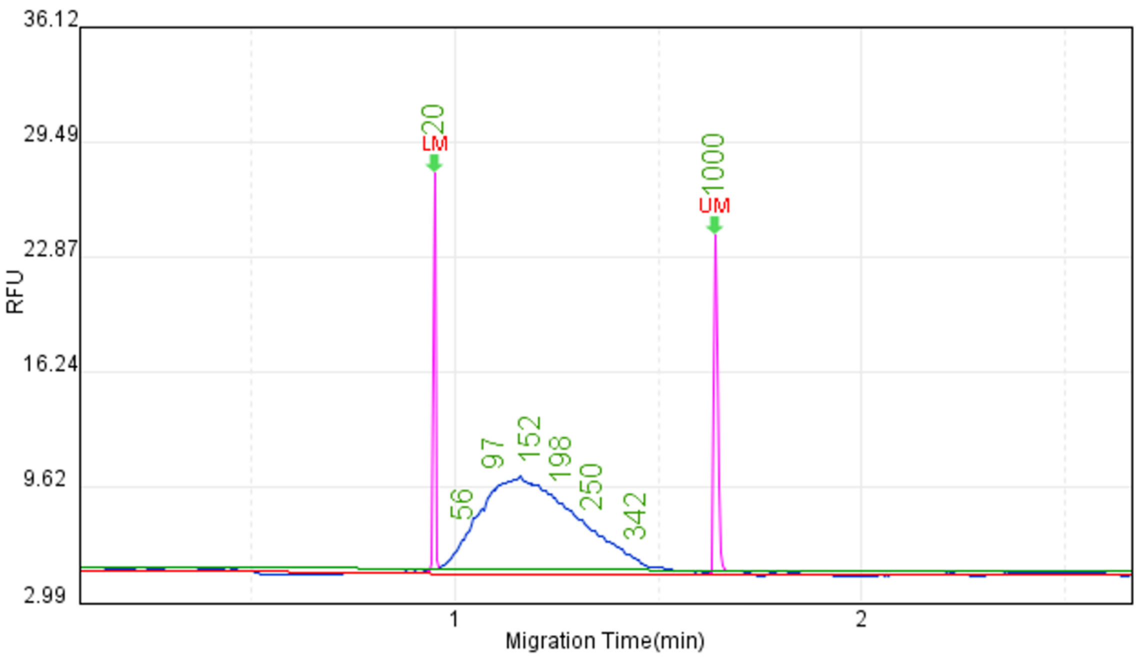

Supplementary Figure 10: Capillary electrophoresis of ultrasonically fragmented DNA using Covaris M220 (power at 75 W for 510 seconds), showing a peak at 150 bp (blue), markers are in pink and automated background calculation is shown in green.

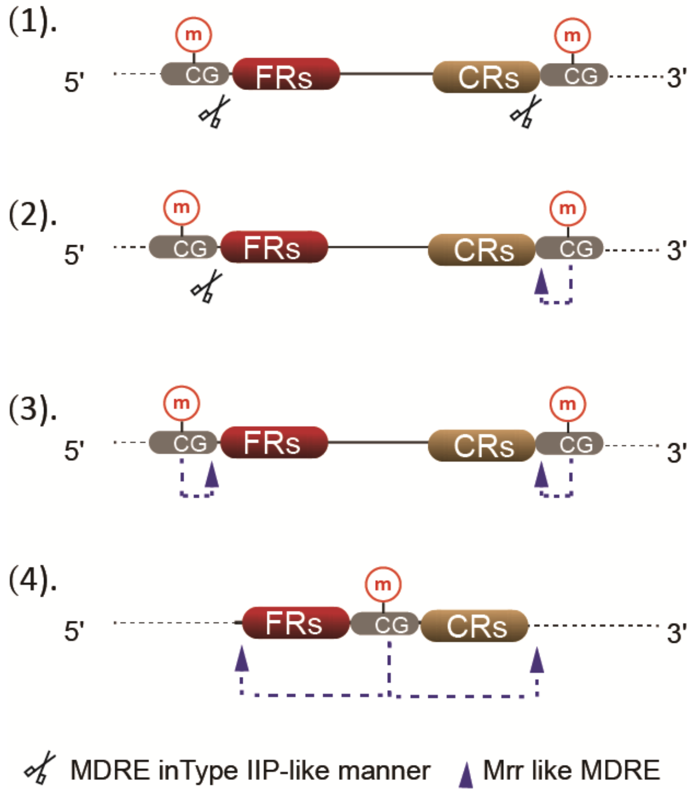

Supplementary Figure 11. Four different digestion scenarios for the generation of P1 with two specific ends generated using different type MDREs: (1) Within Type IIP-like manner at two different locations; (2) Within Type IIP-like and Mrr-like manner independently at two different locations; (3) Within Mrr-like manner independently at two different locations; (4) Within Mrr-like manner at a single location.

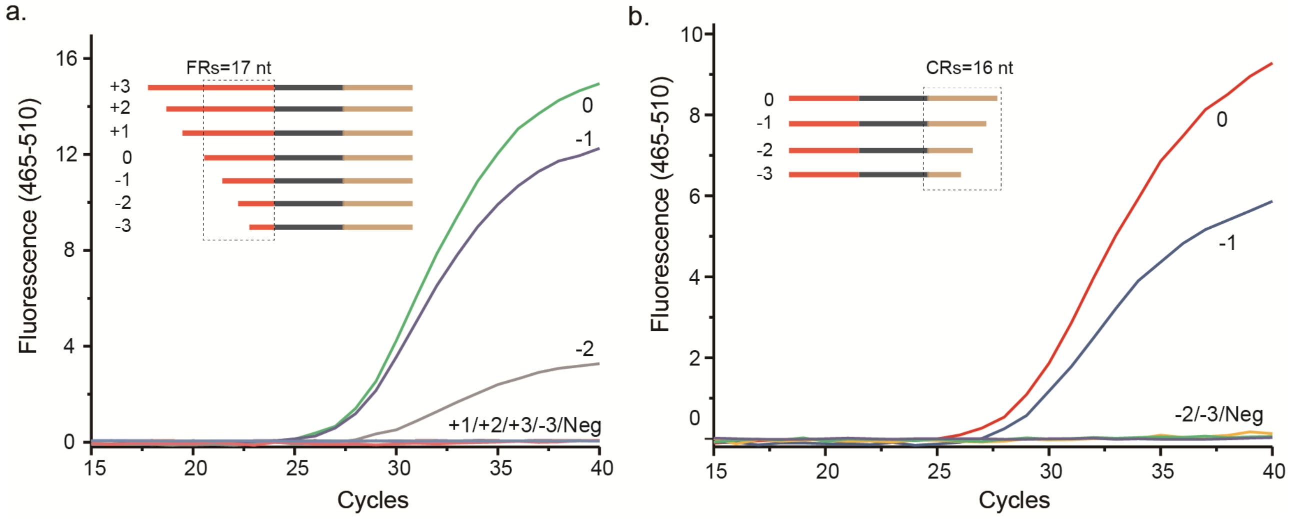

Supplementary Figure 12. The impact of the level of fragmentation on co-methylation detection using the SEPTIN9 amplification system. Different artificial oligos were synthesized and served as template (ca. 20 copies/reaction). (a) The oligos had the same CRs region but different FRs (+3 nt red, +2 blue, +1 orange, 0 green, -1 dark blue, -2 light grey, negative yellow). The base overhung at the 5' end of templates inhibits the amplification, even with only 1 base change. The amplification efficiency decreased as the FRs sizes decreased. (b) The oligos had the same FRs region but different CRs (0 red, -1 dark blue, -2 green, -3 grey, negative yellow). The amplification efficiency decreased as the length of CRs decreased. Source data are provided as a Source Data file.

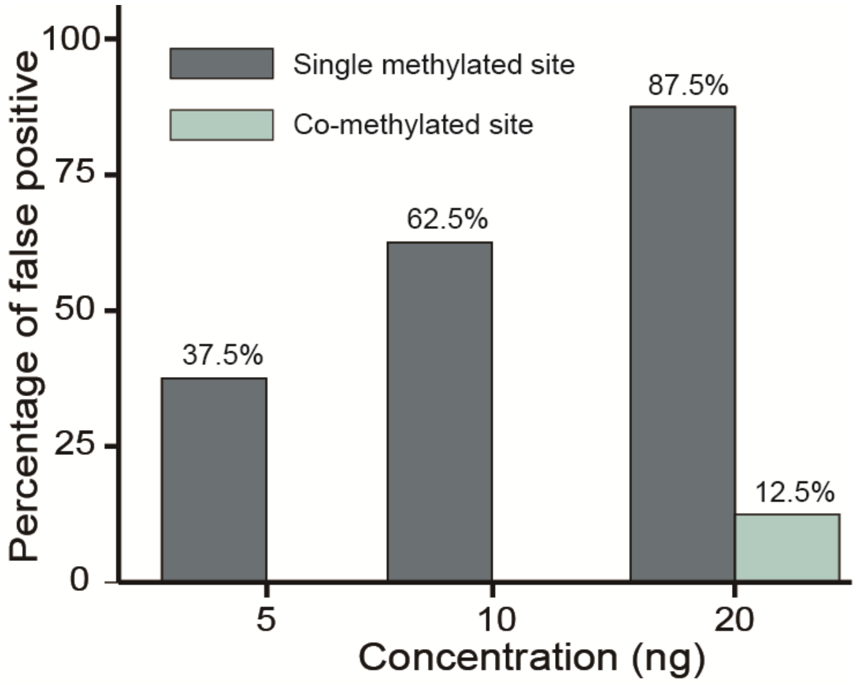

Supplementary Figure 13. Different mechanisms were used to detect 5, 10, and 20 ng fragmented DNA with eight repeats. The amplification systems were described in Figure 2 (single methylated site, grey) and Figure 5 (co-methylated site, pale green), respectively. Concentrations are in ng in each reaction. Source data are provided as a Source Data file.

Supplementary Table 1. Primer sequences for location-specific detection of 5-mC. iSp18 is a PEG18 spacer, equivalent to 6nt length.

Name	Sequence (5'-3')
T1	TTGTGACCC(5mC)GAGTGCACGCCAGGCGAGGCC(5mC)GGATGCTGGGTCCTCCCCGCTTTGGAGCTTCGCTGGCCT CCTTCGGAAGCTCCCCTCCACCCGCAGGAGACAAGACTCCGTGGGT
T2	TTGTGACCC(5mC)GAGTGCACGCCAGGCGAGGCCGGATGCTGGGTCCTCCCCGCTTTGGAGCTTCGCTGGCCTCCTT CGGAAGCTCCCCTCCACCCGCAGGAGACAAGACTCCGTGGGT
T3	TTGTGACCCGAGTGCACGCCAGGCGAGGCC(5mC)GGATGCTGGGTCCTCCCCGCTTTGGAGCTTCGCTGGCCTCCTT CGGAAGCTCCCCTCCACCCGCAGGAGACAAGACTCCGTGGGT
T4	TTGTGACCCGAGTGCACGCCAGGCGAGGCCGGATGCTGGGTCCTCCCCGCTTTGGAGCTTCGCTGGCCTCCTTCGGA AGCTCCCCTCCACCCGCAGGAGACAAGACTCCGTGGGT
TFP1	GCCTGTCAGCCAACGGTATTCATCTTTGGCGAGGCCGGATGC/iSp18/TCCTGCGGGTGGAGGGGAGCT
Cut helper1	CGCCTGGCGTGCACTCGGGTC
TSP1	CCGAAGGAGGCCAGCGAAGC
Probe1	FAM-TCCTCCCCGCTTTGGA-MGB
AP	GCCTGTCAGCCAACGGTATTCATC
TFP2	GCCTGTCAGCCAACGGTATTCATCTTTCTCCCCGCTTTGGAG/iSp18/ACCCACGGAGTCTTGTCTCCTGC
Cut helper2	GAGGACCCAGCATCCGGCC
TSP2	GTGGGTGGAGGGGAGCTTC
Probe	FAM-CTTCGCTGGCCTCCTT-MGB

Supplementary Table 2: Primer sequences for Cleavage scenarios with two mCG at different distances (Figure 5)

Name	Sequence (5'-3')
CpG>12-TFP	GCCTGTCAGCCAACGGTATTCATCGGACGCGCAGCTGG/iSp18/AGGGTCCTCTCCAGCACG
CpG=11/12-TFP	GCCTGTCAGCCAACGGTATTCATCCGAGGCGCAGCTGGA/iSp18/CAGCACGTCCGCGGCCG
CpG<11-TFP	TGTCAGCCAACGGTATTCATCGGGATCATTTCGGAC/iSp18/GGGTCCTCTCCAGCACGTCC
CpG-TSP	CAGCAGCCAGCCCAGCAC
CpG-probe	CCACCTTCGAAGTCCGAA
CpG>12-helper	GCGTCCAGAGCGGGGTCCGACATGA-P
CpG=12-helper	GCCTCGAGCGCGGGTCCGACATGA-P
CpG=11-helper	GCCTCGAGCGCGGGGTCCGACATGA-P
CpG<11-helper	CCCATCCAGCTGCGCGTTGACC-P

Supplementary Table 3. Primer sequences for STEM-PCR for single site methylation. Underlined bases are 2'-O-methyl modified

Name	Sequence (5'-3')
S9TFP	TGTCAGCCAACGGTATTCATCTTTGCGCAGCTGGATGGG/iSp18/GTCCGCGGCCGCAGCA
S9TSP	TGCCAGCCCAGCACCCA
S9D	FAM-CCTTCGAAGTCCGAAATGA-MGB
S9AP	GCCTGTCAGCCAACGGTATTCATC
SFRP2TFP	GCCTGTCAGCCAACGGTATTCATCTTTGCGACCCCGAGGG/iSp18/GGCCCGGGACAAGCTCGAACTC
SFRP2TSP	CGGCCGCCTCGCCCTTC
SFRP2D	VIC-CTCCGCTCCCTCTGC-MGB
SFRP2AP	GCCTGTCAGCCAACGGTATTCATC
TSP	GCCTGTCAGCCAACGGTATTCATC

Supplementary Table 4. Primer sequences for STEM-PCR studies and BS-sequencing.

Function	Name	Sequence (5'-3')
BS-PCR	SFRP2PF1	GGAGAAGTAGGGTTGGTTAAAGAG
	SFRP2PR1	CTACCAACTTTTCGAAACCCC
	S9PF1	GTTAGTGTTGTATTGTAGGAG
	S9PR1	CAACCCAACACCCACCTT
MSP-PCR	SFRP2PF2	GTTTAGGTAGTAGTGCGAGGC
	SFRP2PR2	ACTCCGCTCCCTCTACCC
	S9PF2	TTTTCGCGCGATTCGTTG
	S9PR2	ACCCACCTTCGAAATCCG

Supplementary Table 5. Primer sequences for EGFR L858R detection

Name	Sequence (5'-3')
PNA modified blocker	TTGGGCGGGCCAA
TFP	GAGCGCAGGTCGTACTCGAATCTGTGATCTTGAC/iSp18/CGCAATCAGGCAATACCATAGCAAACACCGCAGCATG
AP1	GAGCGCAGGTCGTACTCG
AP2	CGCAATCAGGCAATACCATAGC
L858R probe	FAM-AATCTGTGATCTTG-MGB

Supplementary Table 6. Primer sequences for co-methylation. Underlined bases are 2’-O-methyl modified

Name	Sequence (5’-3’)
Septin9-1 BP	TGCCGTCAGAGTCCTGTCTCGAGCGACCCGCTGCCCAC-SH C6
Septin9-1 TFP	CGGACCCATGCCAACGGTATTCATCGTTGACCGCGGGGTCCG/iSp18/AGATGTGGCACTGACAATGCCGTCAGAGTCCTGTCTCGA
Septin9-1 AP1	TGCCAACGGTATTCATCGTTGACC
Septin9-1Probe	VIC-CCATCATGTCGGACCC-MGB
Septin9-1 AP2	CGGCGTCAGATGTGGCACTGACAA

Supplementary Table 7. Primer sequences for co-methylation. Underlined bases are 2'-O-methyl modified

Name	Sequence (5'-3')
Septin9-2 BP	TCGCTCGGtCCACCTGTCCCCTGGCTCAGCTT-SH C6
Septin9-2 TFP	TGCTCACAATCATGCCCGCCTTCCTCCCC/iSp18/TCAGATTGGCAGTCACATTCGCTCGGTCCACCTGT
Septin9-2 AP2	TGCTCACAATCATGCCCGCC
Septin9-2 Probe	FAM-TCCCCCATTCATTCAGC-MGB
Septin 9-2 AP1	CGGCGTCAGATTGGCAGTCACAT
Septin 9-T1	GCGCCCGCCTTCCTCCCCCATTCATTCAGCTGAGCCAGGGGGCCTAGGGGCTCCTCCGG
Septin 9-T2	GCGTTGACCGCGGGGTCCGACATGATGGTGCAGCCTACCGATACTCAAGTCCGTCATACTGGTGGGCAGCGGGTCGCGC
Septin 9-T3	GCGTTGACCGCGGGGTCCGACATGATGGTGCAGCCTACCGATACTCAAGTCCGTCATAACCTCGAATGAGTGTGACCTAATTTAGCGACTGGTGGGCAGCGGGTCGCGC
Septin 9-T4	GCGTTGACCGCGGGGTCCGACATGATGGTGCAGCCTACCGATACTCAAGTCCGTCATAACCTCGAATGAGTGAGTCCTAATTTAGCGACGGTAAGCTTCGACCGCTAGCCAAATGTCCCTGGTGGGCAGCGGGTCGCGC
Septin 9-T5	GCGTTGACCGCGGGGTCCGACATGATGGTGCAGCCTACCGATACTCAAGTCCGTCATAACCTCGAATGAGTGAGTCCTAATTTAGCGACGGTAAGCTTCGACCGCTAGCCAAATGTCTCGCAGCACGATAACAGCTACACTGACCTCTCTGGTGGGCAGCGGGTCGCGC

Supplementary Table 8. The results of STEM-PCR and Bisulfite sequencing using cfDNA. Samples 1-9 are healthy controls, samples 10-14 are patients diagnosed with colorectal cancer. In addition to patient-derived samples (1-14), we also included different proportions (10% and 1%) of fragmented methylated DNA with 30 ng unmethylated genomic DNA background to benchmark our bi-sulfite sequencing performance to the wider literature. The age and sex of patients was not recorded.

Sample NO.	STEM-PCR		Bisulfite sequencing	
	input cfDNA (ng)	Ct value	input cfDNA (ng)	Methylation haplotype load
1	1	N	16.1	0.07%
2	1	N	20.9	0.10%
3	1	N	31	0.06%
4	1	N	31	0.07%
5	1	N	26.2	0.05%
6	1	N	31	0.07%
7	1	N	31	0.04%
8	1	N	22.1	0.05%
9	1	N	22.4	0.04%
10	1	N	31	0.43%
11	1	N	31	0.13%
12	1	27.86	31	1.89%
13	1	25.25	31	5.49%
14	1	N	31	0.05%
10%			30	21.28%
1%			30	3.01%
0%			30	0.15%

N: means no amplification
