# Supplementary material for: Sequence terminus dependent PCR for site-specific mutation and modification detection
Source: Nat Commun. 2023 Mar 1;14:1169. doi: 10.1038/s41467-023-36884-4 (PMC9978023; doi:10.1038/s41467-023-36884-4)
Supplement: Supplementary file 3 — Reporting Summary [file 41467_2023_36884_MOESM3_ESM.pdf]

nature portfolio

Corresponding author(s): Hong Xu, Jonathan M. Cooper and Hongchen Gu

Last updated by author(s): Dec 17, 2022

Reporting Summary

Nature Portfolio wishes to improve the reproducibility of the work that we publish. This form provides structure for consistency and transparency in reporting. For further information on Nature Portfolio policies, see our Editorial Policies and the Editorial Policy Checklist.

Statistics

For all statistical analyses, confirm that the following items are present in the figure legend, table legend, main text, or Methods section.

n/a	Confirmed	
☐	☒	The exact sample size (*n*) for each experimental group/condition, given as a discrete number and unit of measurement
☐	☒	A statement on whether measurements were taken from distinct samples or whether the same sample was measured repeatedly
☒	☐	The statistical test(s) used AND whether they are one- or two-sided<br>*Only common tests should be described solely by name; describe more complex techniques in the Methods section.*
☒	☐	A description of all covariates tested
☒	☐	A description of any assumptions or corrections, such as tests of normality and adjustment for multiple comparisons
☐	☒	A full description of the statistical parameters including central tendency (e.g. means) or other basic estimates (e.g. regression coefficient) AND variation (e.g. standard deviation) or associated estimates of uncertainty (e.g. confidence intervals)
☒	☐	For null hypothesis testing, the test statistic (e.g. *F*, *t*, *r*) with confidence intervals, effect sizes, degrees of freedom and *P* value noted<br>*Give P values as exact values whenever suitable.*
☒	☐	For Bayesian analysis, information on the choice of priors and Markov chain Monte Carlo settings
☒	☐	For hierarchical and complex designs, identification of the appropriate level for tests and full reporting of outcomes
☒	☐	Estimates of effect sizes (e.g. Cohen's *d*, Pearson's *r*), indicating how they were calculated

Our web collection on statistics for biologists contains articles on many of the points above.

Software and code

Policy information about availability of computer code

Data collection	real-time amplification curves were obtained using commercial real-time PCR machine software (Lightcycler 480 v1.5; Lightcycler 96 v1.2 and AppliedBiosystems StepOnePlus v2.0)
Data analysis	Microsoft excel 365 was used to analyse the curves with results plotted with Origin (OriginLabs, v2016), Sequencing data was analysed using Chromas (Technelcium Pty, v2.6.6)

For manuscripts utilizing custom algorithms or software that are central to the research but not yet described in published literature, software must be made available to editors and reviewers. We strongly encourage code deposition in a community repository (e.g. GitHub). See the Nature Portfolio guidelines for submitting code & software for further information.

Data

Policy information about availability of data

All manuscripts must include a data availability statement. This statement should provide the following information, where applicable:
- Accession codes, unique identifiers, or web links for publicly available datasets
- A description of any restrictions on data availability
- For clinical datasets or third party data, please ensure that the statement adheres to our policy

The data generated in this study have been deposited in The University of Glasgow repository Enlighten at http://dx.doi.org/10.5525/gla.researchdata.1298. Source data are provided with this paper

Human research participants

Policy information about studies involving human research participants and Sex and Gender in Research.

Reporting on sex and gender	the study focusses on analytical characterisation and gender characteristics are not reported.
Population characteristics	samples were collected from participants when they were in hospital to undergo surgery. The cohort comprised adult patients who had received a clinical diagnosis for colorectal cancer and scheduled to undergo either biopsy or resection surgery. Pregnant women were excluded, along with patients who had received any other form of treatment for colorectal cancer. We did not record age or sex.
Recruitment	Participants were recruited by their treating physician in the hospital upon their scheduled intervention (either biopsy or resection surgery). A nurse or the physician obtained written informed consent prior to enrolment in the study. The collection from surgical patients will potentially lead to bias in the number of positive samples.
Ethics oversight	The study was approved by the Human Research Ethics Committee of Renji Hospital (Shanghai, China) - approval RGH 09/04.

Note that full information on the approval of the study protocol must also be provided in the manuscript.

Field-specific reporting

Please select the one below that is the best fit for your research. If you are not sure, read the appropriate sections before making your selection.

[x] Life sciences [ ] Behavioural & social sciences [ ] Ecological, evolutionary & environmental sciences

For a reference copy of the document with all sections, see nature.com/documents/nr-reporting-summary-flat.pdf

Life sciences study design

All studies must disclose on these points even when the disclosure is negative.

Sample size	no sample size calculation was performed. We aimed to recruit 20 volunteers to establish a robust analytical comparison with the gold standard technique. The number was chosen based on experience in the development of new assays and indeed demonstrated that our technique is able to detect false negatives.
Data exclusions	no data were excluded from analysis
Replication	Data was replicated at lest 3 times
Randomization	the samples were not randomised but processed in the order they were received which is random to all covariates
Blinding	the analytical studies were not carried out blinded but included biological replicates to ensure minimal experimental bias. The analysis of patient samples was carried out single blind: the experimenter conducted both STEM-PCR and BS-seq at the same time and results were compared to clinical evaluation independently (by an investigator different from the experimenter).

Reporting for specific materials, systems and methods

We require information from authors about some types of materials, experimental systems and methods used in many studies. Here, indicate whether each material, system or method listed is relevant to your study. If you are not sure if a list item applies to your research, read the appropriate section before selecting a response.

Materials & experimental systems

n/a	Involved in the study	
[x]	[ ]	Antibodies
[ ]	[x]	Eukaryotic cell lines
[x]	[ ]	Palaeontology and archaeology
[x]	[ ]	Animals and other organisms
[x]	[ ]	Clinical data
[x]	[ ]	Dual use research of concern

Methods

n/a	Involved in the study	
[x]	[ ]	ChIP-seq
[x]	[ ]	Flow cytometry
[x]	[ ]	MRI-based neuroimaging

Eukaryotic cell lines

Policy information about cell lines and Sex and Gender in Research

Cell line source(s)	Hela Cells (ATCC, cat # 30-2003)
Authentication	None of the cell lines were authenticated. They were obtained commercially from trusted suppliers.
Mycoplasma contamination	the cell lines were not tested for mycoplasma
Commonly misidentified lines (See ICLAC register)	No misidentified cell lines were used.
